# Multi-Input CNN-LSTM deep learning model for fear level classification based on EEG and peripheral physiological signals

**DOI:** 10.3389/fpsyg.2023.1141801

**Published:** 2023-06-01

**Authors:** Nagisa Masuda, Ikuko Eguchi Yairi

**Affiliations:** Graduate School of Science and Engineering, Sophia University, Tokyo, Japan

**Keywords:** fear, emotion recognition, Convolutional Neural Network, long-term and short-term memory, physiological signal, EEG

## Abstract

Objective and accurate classification of fear levels is a socially important task that contributes to developing treatments for Anxiety Disorder, Obsessive–compulsive Disorder, Post-Traumatic Stress Disorder (PTSD), and Phobia. This study examines a deep learning model to automatically estimate human fear levels with high accuracy using multichannel EEG signals and multimodal peripheral physiological signals in the DEAP dataset. The Multi-Input CNN-LSTM classification model combining Convolutional Neural Network (CNN) and Long Sort-Term Memory (LSTM) estimated four fear levels with an accuracy of 98.79% and an F1 score of 99.01% in a 10-fold cross-validation. This study contributes to the following; (1) to present the possibility of recognizing fear emotion with high accuracy using a deep learning model from physiological signals without arbitrary feature extraction or feature selection, (2) to investigate effective deep learning model structures for high-accuracy fear recognition and to propose Multi-Input CNN-LSTM, and (3) to examine the model’s tolerance to individual differences in physiological signals and the possibility of improving accuracy through additional learning.

## 1. Introduction

In recent years, as affective computing (Picard and Healey, 1997), which aims to provide a function to dynamically adjust interaction with a computer according to the user’s emotions, has been attracting attention, the importance of human emotion recognition as a fundamental technology is increasing. Emotion recognition is a technology that can be applied to various fields such as healthcare, education, criminal investigation, marketing, and entertainment, so many experts from multiple fields are conducting research (Chanel et al., 2011; Oh et al., 2017; Bertacchini et al., 2018; Yang et al., 2018; Sajjad et al., 2020). It is expected that the global market for emotion recognition will continue to expand in the future (Markets and Markets, 2017).

Emotion recognition methods can be roughly divided into two types according to the signals used. The first is emotion recognition using external behavioral signals such as speech (Schuller, 2018), facial expressions (Byoung, 2018), and body gestures (Gunes and Piccardi, 2007). This method has the advantage that it is easy to collect signal data because it is possible to use common devices embedded in PCs and smartphones, and open data containing such information exist on the Internet (Sidorova, 2007; Liu et al., 2015; Yoon et al., 2019). Emotion recognition accuracy using external behavioral signals is improving year by year by introducing deep learning to these collected large amounts of signal data. However, emotion recognition based on external behavioral signals has problems with privacy and the installation location of the device.

The second is emotion recognition using internal physiological signals. Internal physiological signals include Electroencephalography (EEG), Electromyogram (EMG), Galvanic Skin Response (GSR), Respiration, Temperature, Electrooculogram (EOG), Electrocardiogram (EKG), etc. (Shu et al., 2018; Dzedzickis et al., 2020). Emotion recognition using internal physiological signals is more effective when compared to speech and gestures (Zhang et al., 2020). The method requires expensive equipment and strict measurement conditions, which are not widely used at present, but it is possible to overcome this disadvantage by improving the technology in the future.

We focus on the level classification of fear emotion, one of the research topics of emotion recognition using internal physiological signals. This paper discusses a deep learning model for estimating human fear levels with high accuracy using multichannel EEG and multimodal peripheral physiological signals contained in the DEAP dataset (Koelstra et al., 2012). Objective and accurate classification of fear levels is a socially important task that contributes to developing treatments for Anxiety Disorder, Obsessive–compulsive Disorder, Post-Traumatic Stress Disorder (PTSD), and Phobia. Problems have been reported in treating these diseases that patients’ self-reported fears do not match the treatment, resulting in aggravation of disability and ineffectiveness.

This study aims to approach the above issues and contribute to the following; (1) to present the possibility of recognizing fear emotion with high accuracy using a deep learning model from physiological signals without arbitrary feature extraction or feature selection, (2) to investigate effective deep learning model structures for high-accuracy fear recognition and to propose Multi-Input CNN-LSTM combining Convolutional Neural Network (CNN) and Long Sort-Term Memory (LSTM), and (3) to examine the model’s tolerance to individual differences in physiological signals and the possibility of improving accuracy through additional learning.

## 2. Related work

Many studies in recent years have investigated the Dynamic Difficulty Adjustment (DDA) mechanism in computer games to enable game-playing experiences tailored to individual characteristics (Zohaib, 2018). Several studies have attempted to predict the emotional level of players to adjust to game difficulty. Liu et al. (2009) examined the affective model that recognizes anxiety levels during games. Fifteen participants played the anagram game and the ping pong game and assessed their anxiety using a 9-point Likert scale. The authors collected Photoplethysmography, EKG, Heart Sound, GSR, EMG, and Skin Temperature signals during the games and derived 43 features. Regression Tree, K-Nearest Neighbors (KNN) (Altman, 1992), Bayes Network, and Support Vector Machine (SVM) (Cortes and Vapnik, 1995) were trained using these features and evaluated by Leave-One-Out Cross-Validation (LOO-CV). A Regression Tree-based model yielded an accuracy of 88% offline and 78% real-time with a three-level classification of anxiety. In addition, the effects on the gaming experience were evaluated and compared by applying performance-based DDA and affect-based DDA to the same computer game. The difficulty level varied based on the player’s grades for the performance-based DDA and anxiety levels for the affect-based DDA. The study found that affect-based DDA provides players with better performance and a more rewarding gaming experience. Orozco-Mora et al. (2022) examined the feasibility of distinguishing between different stress levels while playing a virtual reality video game. The authors extracted 15 features from EMG, EKG, and GSR acquired when 27 participants played a shooting game to survive by killing zombie enemies. The used classification algorithms were SVM with a Linear and a Radial Basis Function kernel, KNN, Decision Tree, Random Forest (RF) (Breiman, 2001), and Multi-Layer Perceptron. This study could distinguish four stress levels (three difficulty levels and a resting stage) with an accuracy of 91.80% using KNN in five-fold cross-validation.

Stress recognition research as one of emotional recognition has been attracted historically because stress causes various health problems (Quick et al., 1987) and reduces work performance (Baddeley, 1972). Healey and Picard (2005) present methods for collecting and analyzing physiological signals during real-world driving tasks to determine a driver’s stress level. EKG, EMG, hand and foot GSR, and Respiration were recorded while 24 drives the rest (low level), highway (medium level), and city (high level). The driver’s three stress levels were recognized with an accuracy of 97.40% in LOO-CV using the linear discriminant function with 22 features obtained from these signals. The dataset used in the study is partially available on the website PHYSIONET (Healey and Picard, 2010). Deng et al. (2013) proposed the feature selection method based on the performance and the diversity between two features. The same features as in Healey and Picard (2005) were extracted from the seven complete and three partially incomplete driver datasets in Healey and Picard (2010), and the feature sets were selected by the proposed method. Evaluating feature selection results by the Leave-One-Out index based on combinatorial fusion (Hsu et al., 2006), the highest correct rate was 87.69% for a 5-feature set. The results showed much better performance than when using randomly selected features. Ghaderi et al. (2015) tried to get high accuracy for the different numbers of biological sensors, features, and time intervals. The authors divided the complete data of seven drivers in Healey and Picard (2010) into segments for 100, 200, and 300 s intervals, then extracted 78 features. The best of them selected by the feature selection algorithm of Weka (Witten and Frank, 2005) were classified using SVM and KNN. The high classification result was obtained by KNN with five features from three sensors (Respiration, EMG, hand GSR) for 300 s state, with an accuracy of 99% in cross-validation. Increasing the number of sensors and features used did not improve the accuracy.

Research aimed at treating anxiety disorders such as phobias predicts the degree of anxiety or fear. Providing therapy scenarios according to the individual emotion level makes exposure therapy more effective. Šalkevicius et al. (2019) presented the anxiety level prediction framework for a virtual reality exposure therapy system. The GSR, Blood Volume Pulse, and Skin Temperature signals of 30 participants were collected during the presentation in front of virtual listeners and split into four classes based on the subject’s anxiety level measured by the Subjective Unit of Distress Scale (SUDS) (Wolpe, 1969). The statistical features were extracted from collected signals and performed feature selection by RF. The signal fusion-based SVM classifier achieved an accuracy of 80.1% in Leave-One-Subject-Out (LOSO) and 86.3% in 10 × 10-fold cross-validation. The model outperformed models that used standalone signals. Bălan et al. (2019) presented an approach toward classifying fear levels from physiological recordings stored in the DEAP database using various feature extraction methods, feature selection methods, and machine learning methods. The features were the 32-channel EEG (raw values/Power Spectral Densities in the alpha, beta, and theta frequency ranges/Approximate Entropy/Petrosian Fractal Dimension/Higuchi Fractal Dimension), eight peripheral physiological recordings. The Machine Learning methods applied were four Deep Neural Networks with different numbers of hidden layers and neurons per layer, SVM, RF, Linear Discriminant Analysis, and KNN. The feature selection techniques were Fisher selection, Principal Component Analysis, and Sequential Feature Selector. Training and cross-validating a classifier were repeated 10 times using the dataset divided into 70% training and 30% test, and the average accuracy was calculated for each method. The highest accuracy of 85.74% was obtained using RF based on the alpha, beta, and theta amplitudes with no feature selection. Bălan et al. (2020) proposed a Virtual Reality game that can automatically adjust exposure scenarios according to the level of fear. The acrophobic subjects experienced various scenarios and evaluated their fear level with SUDS by exposing them to different heights of buildings in the real world and virtual environment. Eight participants participated in the preliminary experiment to collect training data, and four in the main experiment to collect test data. With the EEG log-normalized powers of the 16 channels in the alpha, beta, and theta frequencies, the GSR, and Heart Rate signal obtained in the experiment as input, the study predicted the current fear level on four scales and judged the next exposure scenario in real-time by KNN, SVM with Linear kernel, RF, Linear Discriminant Analysis, and Deep Neural Networks with varying numbers of hidden layers and neurons. The used model was the most accurate of the trained models on training data, created by the 10-fold cross-validation. When applying the test user’s data to the classifiers trained on other subjects’ data, the highest accuracy was 52.75% using KNN. In the case of applying each subject’s test data to each classifier trained on the same subject’s data from the preliminary experiment, the highest accuracy was 42.50% using SVM.

Most emotion recognition researches use peripheral physiological signals alone or a combination of EEG and peripheral physiological signals. Peripheral physiological sensors worn on the body have the disadvantage of restricting the user’s movement and clothing. The EEG sensor is suitable for emotion recognition in tasks where movement and clothing are essential. Integrating EEG sensors into hats and headbands can be easily attached and detached. The following studies classify the emotional level using only EEG. To ensure the safety of high-altitude workers, Hu et al. (2018) used a deep CNN to detect the degree of fear of heights. The experiment was conducted in a virtual environment, with 60 participants working at height. The study extracted the energy features from EEG data collected during work, converted it into a two-dimensional spectral image, and used VGG-16 (Simonyan and Zisserman, 2014) to predict the degree of acrophobia on four scales from these images. Validated by 31-fold cross-validation, the result was an accuracy of 88.77%. Wang et al. (2020) used the EEG-based Functional Brain Networks, a complex network based on EEGs, to identify the severity of acrophobia. EEGs were collected from 76 subjects walking on a board hanging from a tree in a virtual environment. The subjects were divided into three groups based on Acrophobia Questionnaire and SUDS scores. The authors obtained Functional Brain Networks by computing the functional connectivity between each pair of channels using Synchronization Likelihood (Stam and Van Dijk, 2002) and used these networks to train five CNNs. The CNN with ResNet (He et al., 2016) performs the best, and accuracy reached 98.46% in six-fold cross-validation. Schaaff and Schultz (2009) investigated two feature extraction approaches to build an emotion recognition system from EEG. Five-channel EEG and scores of pleasure levels measured by Self-Assessment Manikin (Bradley and Lang, 1994) were collected from five subjects using pictures from the International Affective Picture System (IAPS) (Lang et al., 2008) to induce pleasant/unpleasant and neutral. To automatically recognize pleasure levels, SVM with a Radial Basis Function kernel was trained by frequency components ranging from 5 to 40 Hz extracted by the Fast Fourier Transform and the combination features of peak alpha frequency, alpha power, and cross-correlation. For the approach that combines features from peak alpha frequency, alpha power, and cross-correlation, the study obtained an accuracy of 48.89% in LOO-CV.

EEG is often corrupted with artifacts from sources such as the eyes, muscles, and cardiac activity. Therefore, EEG-based emotion classification is required not to blink or move to avoid artifact generation or to detect and remove artifacts. Bălan et al. (2020) removed the artifact in real-time by replacing it with the average value of the data recorded during the previous 5 s when detecting a value negative or exceeding one and one-half than the average value for 5 s. Wang et al. (2020) recorded electrooculograms to remove eye artifacts. Ghosh et al. (2023) proposed a method that can automatically detect and remove artifacts using KNN and LSTM.

Being traditionally used by statistical machine learning models and SVM for emotion recognition, the use of Deep Neural Network models has increased recently due to their high recognition accuracy (Zhang et al., 2020). Although the studies of emotion-level recognition are everything described above as far as we investigated, few of them use the Deep Learning model approach based on Deep Neural Networks. In many cases, the Machine Learning model approach is more accurate than the Deep Learning model approach for emotion-level recognition (Bălan et al., 2019, 2020; Petrescu et al., 2021). However, the Machine Learning model approach is required feature extraction and feature selection to improve accuracy. Although Deep Learning methods can achieve high accuracy without these methods, there is currently little research that has achieved high accuracy in emotion-level classification without feature extraction or feature selection. This study aims to develop a Deep Learning model to estimate fear levels more accurately than other works with automatic feature extraction and feature selection using multichannel EEG signals and multimodal peripheral physiological signals in the DEAP dataset (Koelstra et al., 2012).

## 3. Materials and methods

**Figure 1:**
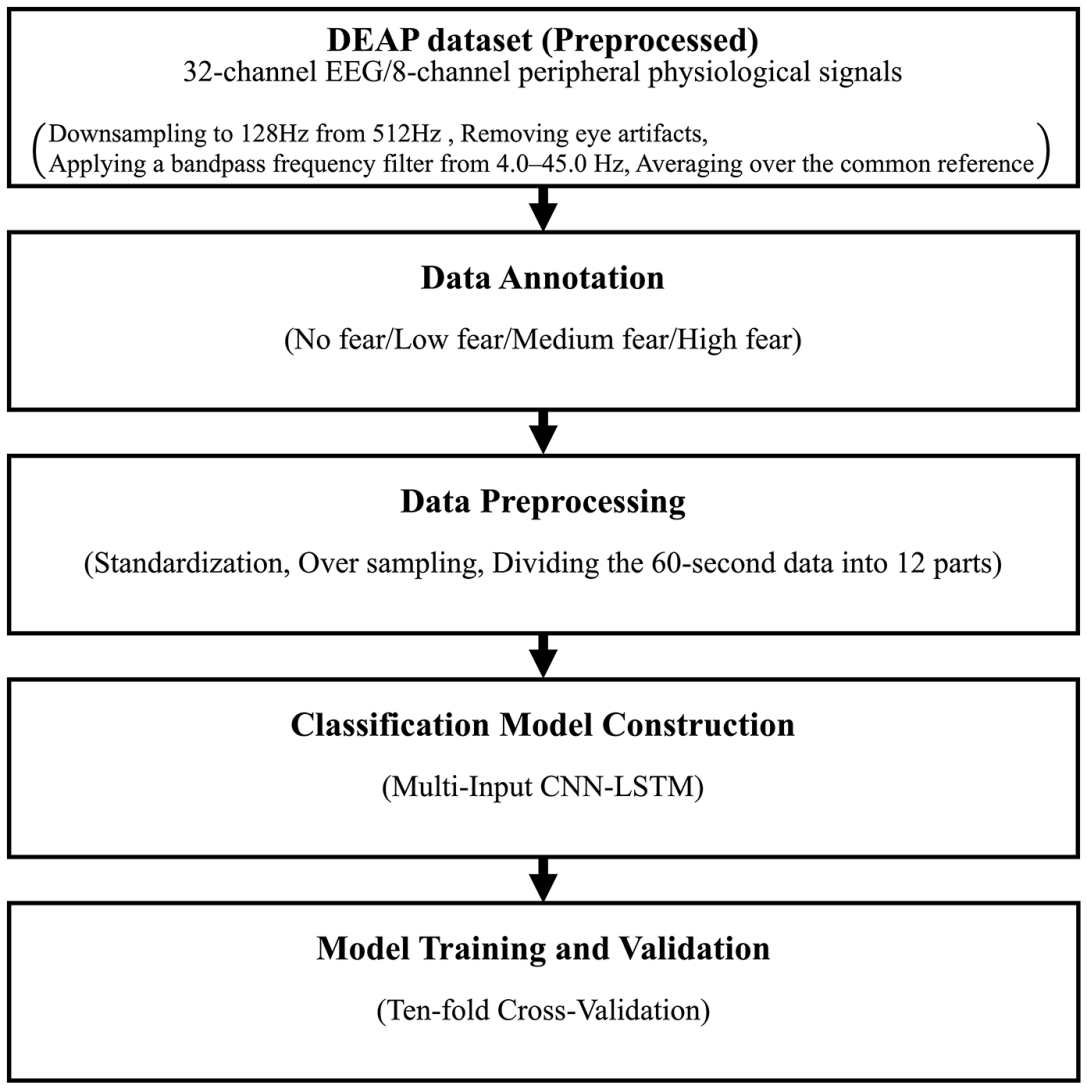
The flowchart of the proposed method.

Figure 1 shows the flowchart of the proposed method. This section is divided into subsections along this figure to explain the details of the proposed method.

### 3.1. DEAP dataset

One of the dimensional models for quantitatively defining emotional states is the Valence-Arousal-Dominance (VAD) model (Russell and Mehrabian, 1977). VAD represents the type of emotion and its intensity by using three numerical value dimensions, valence (displeasure–pleasure), arousal (calmness–excitement), and dominance (being controlled–in control). Fear defines as low valence, high arousal, and low dominance (Demaree et al., 2005). As publicly available datasets containing internal physiological signals for emotion recognition, the most common are ASCERTAIN (Subramanian et al., 2018), DEAP (Koelstra et al., 2012), DECAF (Abadi et al., 2015), MAHNOB-HCI (Soleymani et al., 2012), MIT (Picard et al., 2001), SEEPD-IV (Zheng et al., 2019), and WESAD (Schmidt et al., 2018). Fear level classification requires the dataset to record fear labels by level or labels for valence, arousal, and dominance. The datasets with these labels are DEAP, DECAF, and MAHNOB-HCI. This paper uses the DEAP dataset to classify fear levels because this dataset includes more signals than others. EEG was processed by removing eye artifacts with blind source separation, applying a bandpass frequency filter from 4.0 to 45.0 Hz, and averaging over a common reference.

The DEAP dataset is an open physiological dataset for analyzing human affective states and is used in many studies. This dataset consists of EEG and peripheral physiological signals from 32 participants (50% male and 50% female, 19–37 years old, mean age 26.9) as they watched 40 music videos of 60 s. Participants rated each video in terms of the arousal, valence, liking, and dominance of each video using Self-Assessment Manikin (Bradley and Lang, 1994) on a continuous 9-point scale. The collected physiological signal is 32-channel EEG (FP1, AF3, F3, F7, FC5, FC1, C3, T7, CP5, CP1, P3, P7, PO3, O1, Oz, Pz, FP2, AF4, Fz, F4, F8, FC6, FC2, Cz, C4, T8, CP6, CP2, P4, P8, CP6, CP2, P4, PO4, and O2) and 8-channel peripheral physiological signals. Peripheral physiological signals include EOG (horizontal and vertical), EMG (zygomaticus and trapezius), GSR, Respiration, Plethysmograph, and Skin Temperature, hereafter called PPS. The preprocessed public dataset stores 32 files, one file for each subject, and each file contains the data array of 40 videos × 40 channels (32 EEG + 8 PPS) × 8064 readings and the label array of 40 videos × 4 labels (valence, arousal, dominance, and liking). The data were downsampled to 128 Hz and segmented into 60-s trials, and a 3-s pre-trial baseline was removed. EEG was processed by removing eye artifacts with blind source separation, applying a bandpass frequency filter from 4.0 to 45.0 Hz, and averaging over a common reference.

This paper uses all signals in the DEAP dataset because those are known to be related to fear. When humans feel fear, Blinking Rate (Choi et al., 2015), Heart Rate (detectable by plethysmograph) (Hubert and de Jong-Meyer, 1990; Kometer et al., 2010), Respiration Rate (Harris, 2001), and GSR such as Skin Conductance Response, non-specific Skin Conductance Response Rate, and Skin Conductance Level (James, 1884; Berridge, 1999; Drummond, 1999) increases. Temperature decreases (Dimberg and Thunberg, 2007). In EEG, beta power increases in the left temporal lobe (Kometer et al., 2010; Park et al., 2011). GSR, Heart Rate, and EEG beta waves are essential in predicting fear levels (Bălan et al., 2020).

### 3.2. Data annotation

As mentioned in section 3.1, fear defines as low valence, high arousal, and low dominance. The DEAP dataset is scored on the continuous 9-point scale for each arousal, valence, and dominance, but this study requires the 4-point scale for fear level. Therefore, we relabeled the data based on the following rule (Bălan et al., 2019). Table 1 shows the value of valence, arousal, and dominance labels in the DEAP dataset corresponding to the four-level fear in this study.

**Table 1:** The contrast between the four-level fear in this study (New Label) and the labels for valence, arousal, and dominance in the DEAP dataset (Original Label).

New Label	Original Label		
	Valence	Arousal	Dominance
0: No fear	(7, 9)	(1, 3)	(7, 9)
1: Low fear	(5, 7)	(3, 5)	(5, 7)
2: Medium fear	(3, 5)	(5, 7)	(3, 5)
3: High fear	(1, 3)	(7, 9)	(1, 3)

### 3.3. Data preprocessing

After relabeling, the imbalanced dataset was obtained, which consisted of seven for No fear, 60 for Low fear, 42 for Medium fear, and 35 for High fear. These data were from 28 of the 32 subjects in the DEAP dataset. These subjects had data at any of the four levels, while the remaining four had no data at any level. This dataset is quite unbalanced, but there is currently no balanced open dataset on fear as far as we know. The data numbers were increased from 144 to 1728 by dividing the 60-s data into 12 parts so that each piece of data takes 5 s and 40 channels × 672 readings.

### 3.4. Classification model construction

As fear level classification models, Bălan et al. (2019) used RF with an accuracy of 85.74%, and Bălan et al. (2020) used kNN with an accuracy of 52.75% to classify four levels in cross-validation. The data of both studies is the EEG signal converted to the alpha, beta, and theta frequencies. Petrescu et al. (2021) reported a binary classification of fear with an average accuracy of 92.40% during 10 repeated 7:3 holdouts by reducing the dimensionality with Principal Component Analysis of the GSR and Plethysmographs of the DEAP dataset for inputting to SVM. With the MANHOB dataset (Soleymani et al., 2012), Miranda et al. (2021) performed binary classification of fear with an accuracy of 96.33% with five-fold cross-validation using a classifier composed of SVM and kNN, whose inputs were temporal, frequency, and non-linear based features derived from ECG, Skin Temperature, and GSR signals of only female participants of the dataset.

This study tries to input the raw EEG and PPS into a Deep Learning model and classify them in four classes. Alhagry et al. (2017) recognized low/high arousal, valence, and liking from raw EEG signals stored in the DEAP dataset with high accuracy using LSTM. This paper does not describe the conversion of the input data, and it is thought that the data was input to the LSTM in its original format. Following this result, this study adopted LSTM as a classification model and input data in the same original format but could not obtain sufficient classification accuracy. To solve this problem, we introduced a deep CNN at the pre-input stage of the LSTM. Bălan et al. (2019) reduced data readings from 672 to 1 by the arithmetic mean before inputting it into the RF model. In our proposed method, the 2D Average Pooling layer placed after the 2D Convolutional layer has a similar role. In the research on using accelerometers to recognize human activity (Zeng et al., 2014), the three accelerometer channels (in the X, Y, and Z axes) are input to different Convolutional layers. Following this method, EEG and PPS were input separately.

**Figure 2:**
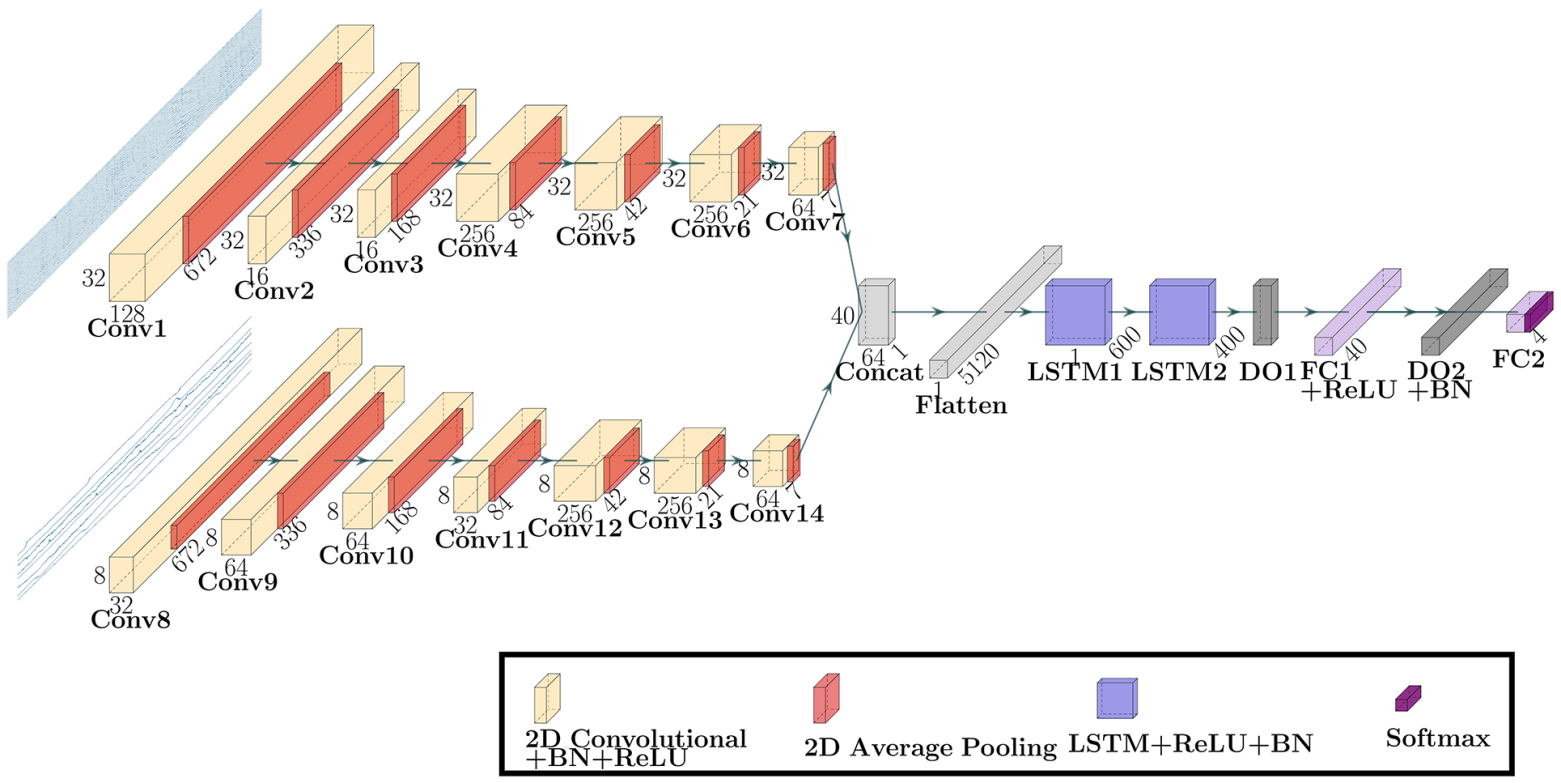
The network architecture of the Multi-Input CNN-LSTM model proposed in this paper. BN is the Batch Normalization, DO is the Dropout, and FC is the Fully Connected.

Figure 2 shows the network architecture of the Multi-Input CNN-LSTM model proposed in this paper. This network was a model that combined two CNNs and one LSTM and comprised two input layers，2 × 7 Convolutional units，2 × 2 LSTM units， a Concatenate layer, a Flatten layer, a Fully Connected layer, and an output layer. The first CNN and the second CNN were entered with 632 × 32 × 1 EEG data and 632 × 8 × 1 PPS data, respectively. The Convolutional unit consisted of a 2D Convolutional layer, a Batch Normalization layer (Loffe and Szegedy, 2015; Li et al., 2019), a Rectified Linear Unit (ReLU) function (Nair and Hinton, 2010), and a 2D Average Pooling layer (Scherer et al., 2010). The kernel sizes were 9 × 1, 9 × 1, 7 × 1, 3 × 1, 3 × 1, 5 × 1, 5 × 1 in the first CNN, and 5 × 1, 9 × 1, 5 × 1, 7 × 1, 7 × 1, 5 × 1, 7 × 1 in the second CNN. In all Convolutional layers, the strides were 1 × 1 and the padding was ‘same.’ The 2D Convolution layer repeats the process of sliding filters in the direction of the readings axis across the input and converting all values in the field to which the filter is applied to a single value by convolution and then creating a feature map that summarizes the presence of the detected feature. The 2D Average Pooling layers downsample the data from 672 to 1 by applying a sliding window processing in the direction of the readings axis to each feature map obtained by convolutional and averaging each feature map within the window. This operation yields 1 reading × 64 filters (because the last convolutional layer has 64 filters) per channel. The LSTM unit consisted of a Bidirectional LSTM (Bi-LSTM) layer (Graves and Schmidhuber, 2005) with one timestep and a ReLU function. LSTM has a memory cell that stores long-term memory and three gates that process based on the certain-time input and the output from the hidden layer that received the previous-time input: the input, forget, and output gate. The input gate determines new information stored in the memory cell, the output gate determines the output value, and the forget gate determines the information discarded from the memory cell. This process classifies the features extracted by CNN. The Deep Learning model was constructed using Keras (Keras Documentation, 2015) library.

### 3.5. Model training and validation

Bălan et al. (2019) used 10-fold cross-validation for the Deep Learning models. To compare their models, we selected 10-fold cross-validation for validating our proposed model. In 10-fold cross-validation, all data was divided into 10 dataset groups. One dataset was selected for the trained model evaluation. The rest of the nine were used for the model training. This process was repeated for all 10 datasets and calculated the average accuracy. Each dataset was standardized for each channel by subtracting the median and dividing by the difference between the data in the top 10% and the bottom 10%. The scikit-learn library (Pedregosa et al., 2011) was used for standardization. As mentioned earlier, the data used in this paper is imbalanced. When the class ratio is unbalanced, data in the minority class is hard to predict. In training data, the data in the minority classes were oversampled using Synthetic Minority Over-sampling Technique (SMOTE) (Chawla et al., 2002) to fit the number of data in the majority class. The number of training and test data per dataset was 2592 and 172–173. The training data was split into 80% training data and 20% validation data in a stratified fashion and was input into the training model. The Adaptive Moment Estimation (ADAM) (Diederik and Kingma, 2015) was used as an optimizer. The number of epochs and batch size were set to 300 and 16. If the categorical cross-entropy loss of validation data stopped decreasing during 15 epochs, the learning rate was reduced by 1/5. If it stopped decreasing during 40 epochs, the network stopped training.

**Figure 3:**
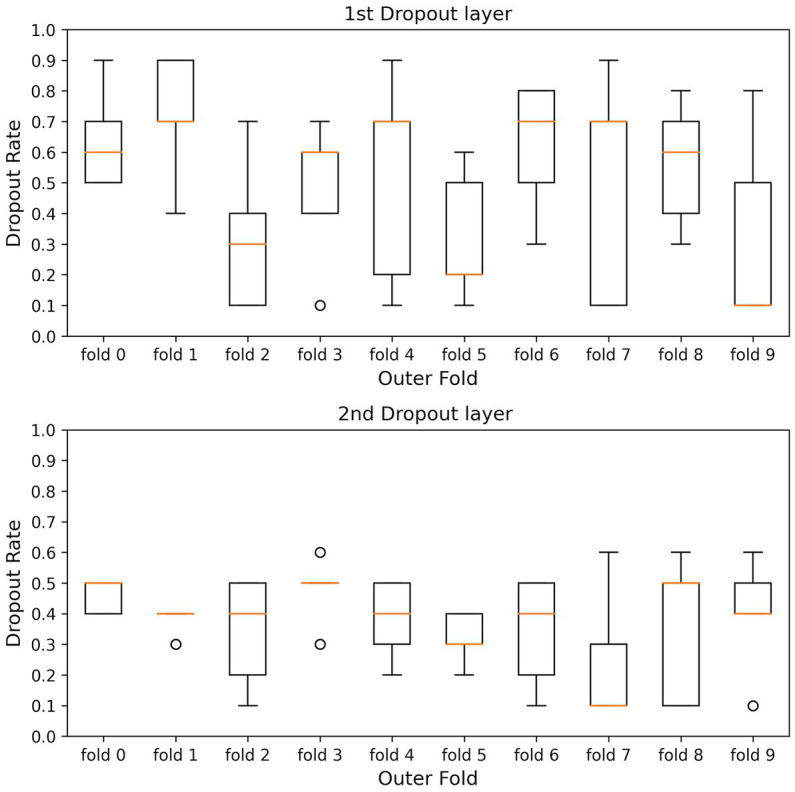
Shows the box plots of the dropout rate of the Dropout layers. 1st and 2nd Drop layers are DO1 and DO2 in Figure 2. The horizontal axis is the outer hold, and the vertical axis is the dropout rate. The values of five inner holds for each outer hold are plotted in each box.

**Figure 4:**
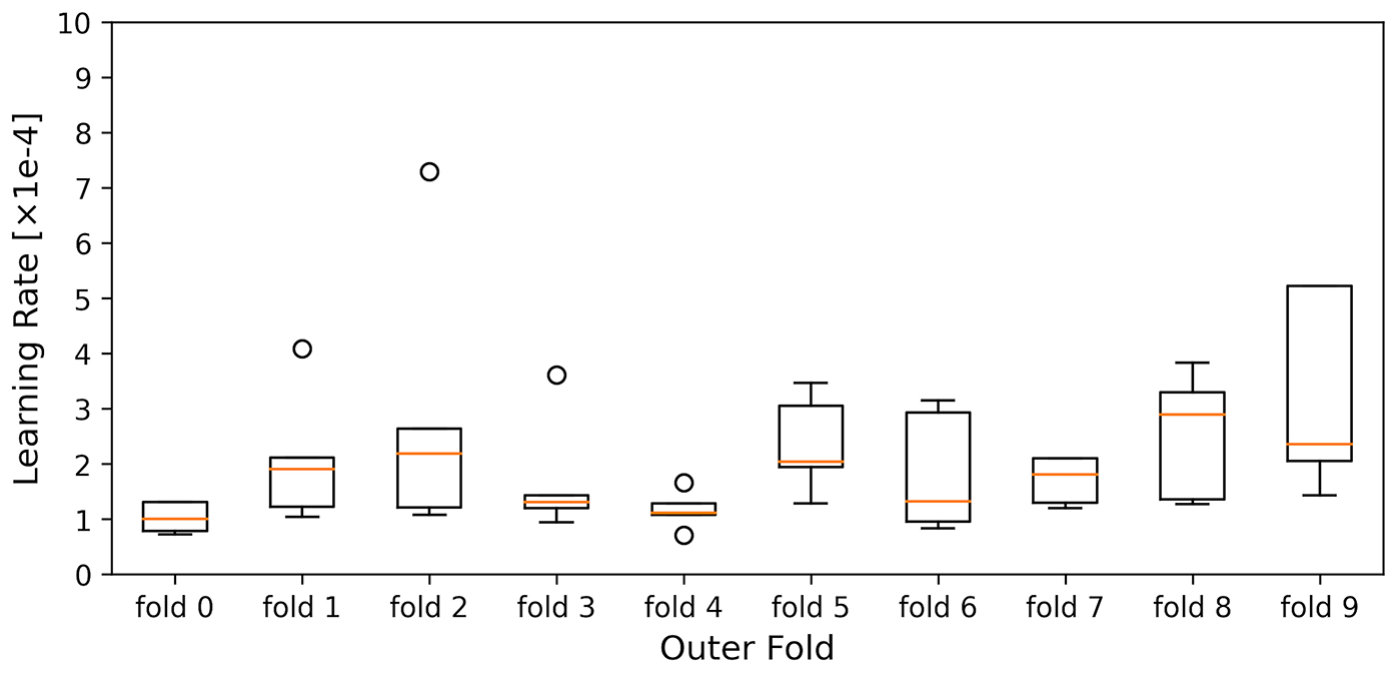
Shows the box plots of the learning rate. The horizontal axis is the outer hold, and the vertical axis is the learning rate. The values of five inner holds for each outer hold are plotted in each box.

The kernel size, the filter size, the number of units in the LSTM layer and fully connected layer, and the dropout rate were optimized to maximize the average accuracy for all folds (models) in the 10-fold cross-validation. The dataset used in this study is highly imbalanced in the fear-level classification tasks. Overfitting is a significant problem in the Deep Neural Network, especially using an imbalanced dataset. To avoid overfitting, the dropout rates of the dropout layers were optimized for each inner fold by dividing each outer fold in 10-fold cross-validation into inner folds. The parameters of the inner hold that achieved the highest accuracy of inner holds for each outer fold were applied to each outer fold model. Figures 3, 4 show the box plots of the dropout rates of the Dropout layers and the learning rate when the number of inner folds is five. The values of five inner holds for each outer hold are plotted in each box. These results indicate that at least 1728 fear data used in this paper in the DEAP dataset have not only class imbalances and also high variability. The hyperparameter optimization was determined using Optuna (Akiba et al., 2019). Ultimately, the hyperparameters obtained when the outer fold was 10 (10-fold cross-validation) and the inner fold was three were applied to the model since the accuracy was highest in the three inner folds.

## 4. Results and discussion

This paper uses accuracy and macro-averaged F1 score as the metrics to evaluate the proposed model performance. The result is rounded off to the third decimal place.

### 4.1. Model design

Tables 2–4 show the network architecture of models tried in this paper and the verification results. This section fixes hyperparameters in all models for performance comparison. The accuracy of LSTM in Table 2 and CNN-LSTM in Table 3 indicates that feature extraction using Convolutional layers and Average Pooling layers improves the accuracy. Multi-Input CNN-LSTM using Max Pooling layers instead of Average Pooling layers results in an accuracy of 86.51% and an F1 score of 86.93%. Average Pooling extracts features more smoothly than Max Pooling, which is presumed to improve accuracy by reducing noise in the data. LSTM scores a better accuracy rate than Multilayer Perceptron (MLP). The result supports that LSTM is effective for emotion classification using physiological signals. LSTM in this paper sets the timestep parameter as ‘1,’ and the LSTM advantage of learning temporal correlations from several sequential data inputs does not work in such a case. Table 2 shows that LSTM, without this advantage, worked the same way as MLP but could train better because of the role of the input and output gates operating between layers. The recognition accuracies in Tables 2, 3 were the best score with the effort of changing the number of features (average values) to be calculated by dividing 672 input readings of LSTM and the effort of changing the number of output readings of the final layer of CNN. A comparison between MLP and CNN from Tables 2, 3 shows that CNN improves the accuracy without LSTM. The CNN with Average Pooling layers performs a filter bank signal decomposition on the raw EEG signal, like or more expressive than an Fast Fourier Transform (FFT).

**Table 2:** The network architecture of MLP and LSTM models tried in this paper.

Model	Layer	Output shape	Activation	With BN	Accuracy	F1 score
MLP	Input	1 × 40		FALSE	88.48	86.86
	Dense	1 × 800	ReLU	TRUE		
	Dense	400	ReLU	TRUE		
	Dense	4	Softmax	FALSE		
LSTM	Input	1 × 40		FALSE	89.41	88.74
	Bi-LSTM	1 × 800	ReLU	TRUE		
	Bi-LSTM	400	ReLU	TRUE		
	Dense	4	Softmax	FALSE		

Conv-AP is the 2D Convolutional layer and 2D Average Pooling layer, and BN is the Batch Normalization layer. The kernel sizes of 2D Convolutional layers are 9 × 1.

**Table 3:** The network architecture of CNN and CNN-LSTM models tried in this paper.

Model	Layer	Output shape	Activation	With BN	Pool size	Accuracy	F1 score
CNN	Input	672 × 40 × 1		FALSE		95.14	95.03
	Conv-AP	336 × 40 × 16	ReLU	TRUE	2 × 1		
	Conv-AP	168 × 40 × 32	ReLU	TRUE	2 × 1		
	Conv-AP	84 × 40 × 64	ReLU	TRUE	2 × 1		
	Conv-AP	42 × 40 × 128	ReLU	TRUE	2 × 1		
	Conv-AP	21 × 40 × 128	ReLU	TRUE	2 × 1		
	Conv-AP	7 × 40 × 128	ReLU	TRUE	3 × 1		
	Conv-AP	1 × 40 × 128	ReLU	TRUE	7 × 1		
	Flatten	1 × 5120		FALSE			
	Dense	1 × 800	ReLU	TRUE			
	Dense	400	ReLU	TRUE			
	Dropout	400		FALSE			
	Dense	40	ReLU	FALSE			
	Dropout	40		TRUE			
	Dense	4	Softmax	FALSE			
CNN-LSTM	Input	672 × 40 × 1		FALSE		96.12	96.23
	Input	672 × 40 × 1		FALSE			
	Conv-AP	336 × 40 × 16	ReLU	TRUE	2 × 1		
	Conv-AP	168 × 40 × 32	ReLU	TRUE	2 × 1		
	Conv-AP	84 × 40 × 64	ReLU	TRUE	2 × 1		
	Conv-AP	42 × 40 × 128	ReLU	TRUE	2 × 1		
	Conv-AP	21 × 40 × 128	ReLU	TRUE	2 × 1		
	Conv-AP	7 × 40 × 128	ReLU	TRUE	3 × 1		
	Conv-AP	1 × 40 × 128	ReLU	TRUE	7 × 1		
	Flatten	1 × 5120		FALSE			
	Bi-LSTM	1 × 800	ReLU	TRUE			
	Bi-LSTM	400	ReLU	TRUE			
	Dropout	400		FALSE			
	Dense	40	ReLU	FALSE			
	Dropout	40		TRUE			
	Dense	4	Softmax	FALSE			

Conv-AP is the 2D Convolutional layer and 2D Average Pooling layer, and BN is the Batch Normalization layer. The kernel sizes of 2D Convolutional layers are 9 × 1.

**Table 4:** The network architecture of Multi-Input CNN and CNN-LSTM models tried in this paper.

Model	Layer	Output shape	Activation	With BN	Pool size	Accuracy	F1 score
CNN	Input	672 × [32/8] × 1		FALSE		96.99	96.66
	Conv-AP	336 × [32/8] × 16	ReLU	TRUE	2 × 1		
	Conv-AP	168 × [32/8] × 32	ReLU	TRUE	2 × 1		
	Conv-AP	84 × [32/8] × 64	ReLU	TRUE	2 × 1		
	Conv-AP	42 × [32/8] × 128	ReLU	TRUE	2 × 1		
	Conv-AP	21 × [32/8] × 128	ReLU	TRUE	2 × 1		
	Conv-AP	7 × [32/8] × 128	ReLU	TRUE	3 × 1		
	Conv-AP	1 × [32/8] × 128	ReLU	TRUE	7 × 1		
	Concatenate	1 × 40 × 128		FALSE			
	Flatten	1 × 5120		FALSE			
	Dense	1 × 800	ReLU	TRUE			
	Dense	400	ReLU	TRUE			
	Dropout	400		FALSE			
	Dense	40	ReLU	FALSE			
	Dropout	40		TRUE			
	Dense	4	Softmax	FALSE			
CNN-LSTM	Input	672 × [32/8] × 1		FALSE		97.97	98.04
	Conv-AP	336 × [32/8] × 16	ReLU	TRUE	2 × 1		
	Conv-AP	168 × [32/8] × 32	ReLU	TRUE	2 × 1		
	Conv-AP	84 × [32/8] × 64	ReLU	TRUE	2 × 1		
	Conv-AP	42 × [32/8] × 128	ReLU	TRUE	2 × 1		
	Conv-AP	21 × [32/8] × 128	ReLU	TRUE	2 × 1		
	Conv-AP	7 × [32/8] × 128	ReLU	TRUE	3 × 1		
	Conv-AP	1 × [32/8] × 128	ReLU	TRUE	7 × 1		
	Concatenate	1 × 40 × 128		FALSE			
	Flatten	1 × 5120		FALSE			
	Bi-LSTM	1 × 800	ReLU	TRUE			
	Bi-LSTM	400	ReLU	TRUE			
	Dropout	400		FALSE			
	Dense	40	ReLU	FALSE			
	Dropout	40		TRUE			
	Dense	4	Softmax	FALSE			

Conv-AP is the 2D Convolutional layer and 2D Average Pooling layer, and BN is the Batch Normalization layer. The kernel sizes of 2D Convolutional layers are 9 × 1.

Tables 2, 3 applies all physiological signals of 40 channels in the DEAP dataset for classification evaluation to this point. When inputting only 32-channel EEG without 8-channel PPS, CNN-LSTM scores the higher F1 score of 97.91%. Inputting only PPS to CNN-LSTM is an F1 score of 89.22%. At first glance, these results show that using PPS causes a loss of accuracy. This paper considered that feature extraction from all physiological signals, including both EEG and PPS, would be the key to improving the accuracy of fear emotion recognition. Our trial and error to improve accuracy led to finding a way to improve accuracy over using EEG alone, in which the model splits 32-channel EEG and 8-channel PPS into two CNNs and then merges them into one LSTM. The recognition accuracies of the Multi-Input CNN-LSTM and Multi-Input CNN in Table 4 were an F1 score of 98.04 and 96.66% in order. These results demonstrate the effectiveness of multi-input for emotion recognition model design.

### 4.2. Comparison of classification accuracies using three evaluation methods

**Figure 5:**
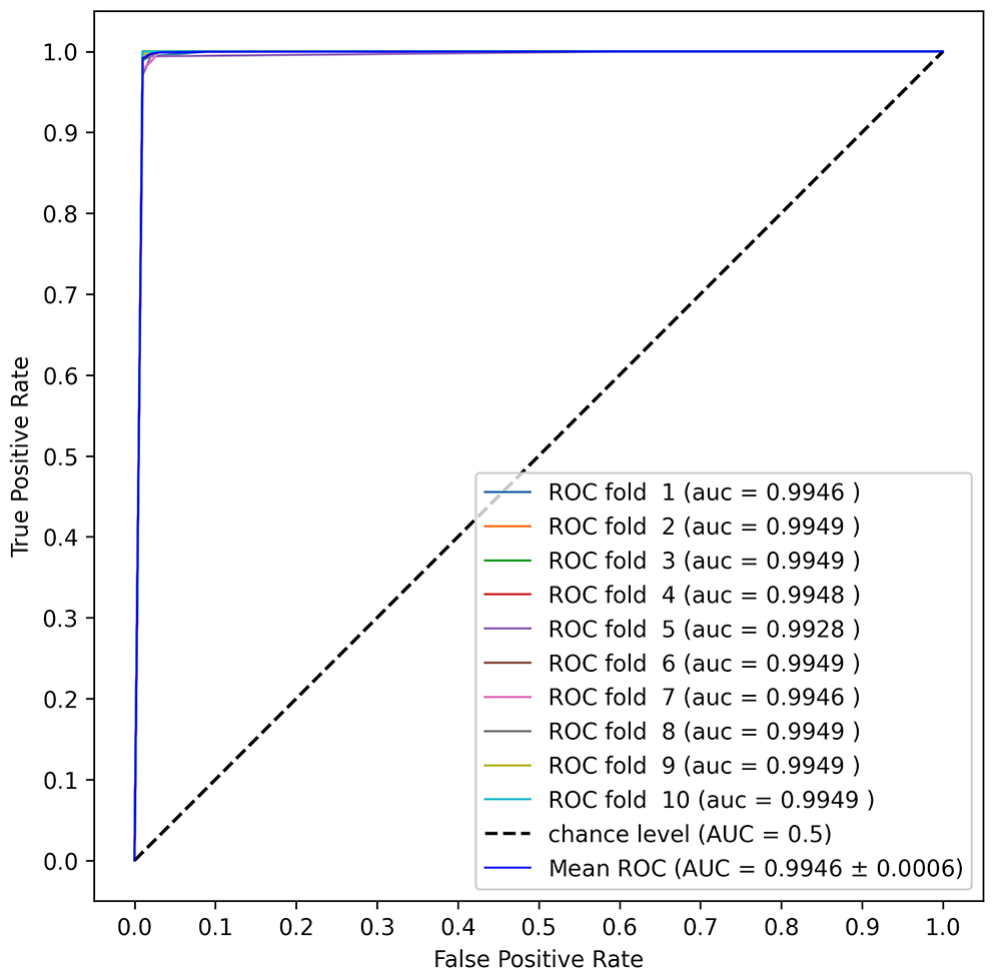
The Receiver Operating Characteristic (ROC) Curve and Area Under the Curve (AUC) values of the Multi-Input CNN-LSTM model proposed in this paper.

Table 5 shows the comparison results between our proposed and previous studies’ methods in cross-validation. The Multi-Input CNN-LSTM achieved an accuracy of 98.79% and an F1 score of 99.01% for the four-level fear classification. These results were obtained when the inner hold was set to three. The accuracy was highest in three inner folds, and the result in five inner folds mentioned in section 3.5 was an accuracy of 98.15% and an F1 score of 98.29%. Optuna, a hyperparameter optimization tool, was used to avoid overfitting, which can be problematic with unbalanced datasets. Our model is about 13% more accurate than the best model RF in Bălan et al. (2019) and 14% more accurate in conditions without feature extraction. Even the simple Deep Neural Network (DNN) (MLP in Table 2) we constructed is more accurate than previous studies’ DNN. Figure 5 shows the Receiver Operating Characteristic (ROC) Curve and Area Under the Curve (AUC) values of the Multi-Input CNN-LSTM model proposed in this paper.

**Table 5:** The comparison between our proposed and previous studies’ methods in cross-validation.

Method	Feature extraction	Accuracy	F1 score
Multi-Input CNN-LSTM (Ours)	–	98.79	99.01
RF (Bălan et al., 2019)	–	84.01	83.85
RF (Bălan et al., 2019)	Alpha, beta and theta Power Spectral Densitys	85.74	85.33
DNN (Ours)	–	85.24	83.00
DNN (Bălan et al., 2019)	Alpha, beta and theta Power Spectral Densitys	68.98	68.46

DNN (Ours) is MLP in Table 2.

K-fold cross-validation cannot confirm how accurately to predict the unknown user’s fear level because data inclusion of the same person in the training and test data is allowed. Although this method evaluates the model performance when the user provides enough training data, assuming the incorporation of this model into applications, it is hard to collect much data on new users for training the model before they use the application.

Therefore, this paper evaluated our proposed system using Leave-One-Subject-Out cross-validation (LOSO), a user-independent verification method, and Calibration, a unique method based on LOSO that evaluates when assuming the actual use start case of a new user.

In LOSO, one of the 28 subjects’ data is selected for the trained model evaluation, while the rest subjects’ data is for the model training. The training data was split into 80% training data and 20% validation data in a stratified fashion as in the 10-fold cross-validation. This process is repeated until all subjects are selected. Table 6 shows the number of test data, accuracy, and F1 score per test subject in LOSO. The result of the average value calculation is an accuracy of 27.77% and an F1 score of 16.50%, a significant decrease from the 10-fold cross-validation result. Since the training data is for 27 subjects, the model should learn all class features no matter who is the test subject.

**Table 6:** The number of test data, accuracy, and F1 score per test subject in LOSO for four-level classification.

**Subject**	**Number of Data**					**Accuracy**	**F1 score**
	**0**	**1**	**2**	**3**	**Total**		
1	12	0	0	0	12	0.00	0.00
2	12	12	12	48	84	21.43	16.07
3	0	48	12	0	60	20.00	13.16
4	0	12	12	0	24	45.83	29.33
5	0	0	12	0	12	0.00	0.00
6	0	24	48	0	72	41.67	27.95
7	0	12	12	12	36	22.22	16.00
8	0	48	12	0	60	5.00	2.94
9	0	24	36	12	72	26.39	18.21
10	0	24	12	0	36	33.33	18.60
11	12	24	0	48	84	14.29	8.11
12	0	0	12	36	48	54.17	26.32
13	0	12	0	60	72	0.00	0.00
14	0	12	24	0	36	33.33	31.43
15	12	36	36	12	96	20.83	15.28
16	0	12	0	24	36	27.78	18.96
17	0	84	36	0	120	38.33	14.11
18	0	60	0	0	60	80.00	44.44
19	0	12	0	0	12	66.67	40.00
20	0	24	48	0	72	0.00	0.00
21	0	24	60	0	84	29.76	14.98
22	0	48	0	24	72	41.67	26.15
23	24	0	0	0	24	0.00	0.00
24	0	12	96	36	144	10.42	9.91
26	0	60	0	0	60	55.00	17.74
29	0	12	0	48	60	41.67	19.60
30	0	48	24	0	72	18.06	12.63
31	12	36	0	60	108	29.63	20.02
Total	84	720	504	420	1728		
Average						27.77	16.50

Accuracy and F1 score are shown as a percentage.

Therefore, the cause of the accuracy loss seems to be that the test data has different characteristics from the training data due to individual differences in the data. If the model learns even a few properties of the test subjects’ data, this problem could be solved.

Calibration extracts partial data from the test dataset of a selected evaluation subject and adds the extracted data to the training dataset of LOSO, the rest of the 27 subjects. This paper added 20 and 40% of each test dataset to the training data. The training data was split into 80% training data and 20% validation data in a stratified fashion. Due to the difference in the data for each subject, the number of additional train data varied from 3 to 29 in the 20% case and from 5 to 58 in the 40% case. This data addition is equivalent to setting up the model by showing a music video to new users for 15–145 s in the 20% case and 25–290 s in the 40% case. Tables 7, 8 show the number of test data, accuracy, and F1 score per test subject in Calibration 20% (Cal20) and Calibration 40% (Cal40) in order. From Tables 7, 8, the classification result was an accuracy of 90.89%/95.07% and an F1 score of 82.05%/94.24% at 20%/40%, which significantly improved. As a result of the model learning the test subject’s data and accommodating individual differences, Cal20 was more accurate than LOSO. Cal40 further improved accuracy by training more data than Cal20. The LOSO result indicates that our proposed model needs help estimating the fear level of new users. The Calibration results show that tens of seconds of user setup before use can improve estimation accuracy significantly.

**Table 7:** The number of test data, accuracy, and F1 score per test subject in Calibration 20% for four-level classification.

Subject	Number of data					Accuracy	F1 score
	0	1	2	3	Total		
1	9	0	0	0	9	100.00	100.00
2	9	10	10	38	67	94.03	89.20
3	0	38	10	0	48	97.92	66.22
4	0	9	10	0	19	100.00	100.00
5	0	0	9	0	9	100.00	100.00
6	0	19	38	0	57	73.68	72.85
7	0	9	9	10	28	89.29	89.28
8	0	38	10	0	48	87.50	45.58
9	0	19	28	10	57	87.72	86.83
10	0	19	9	0	28	100.00	100.00
11	10	19	0	38	67	74.63	53.85
12	0	0	9	29	38	100.00	100.00
13	0	9	0	48	57	98.25	66.32
14	0	9	19	0	28	67.86	65.71
15	9	28	29	10	76	90.79	90.21
16	0	9	0	19	28	100.00	100.00
17	0	67	29	0	96	86.46	84.38
18	0	48	0	0	48	100.00	100.00
19	0	9	0	0	9	100.00	100.00
20	0	19	38	0	57	70.18	45.99
21	0	19	48	0	67	85.07	77.43
22	0	38	0	19	57	85.96	81.90
23	19	0	0	0	19	94.74	48.65
24	0	9	77	29	115	78.26	50.31
26	0	48	0	0	48	100.00	100.00
29	0	10	0	38	48	100.00	100.00
30	0	38	19	0	57	85.96	85.42
31	9	29	0	48	86	96.51	97.17
Total	65	569	401	336	1371		
Average						90.89	82.05

Accuracy and F1 score are shown as a percentage.

**Table 8:** The number of test data, accuracy, and F1 score per test subject in Calibration 40% for four-level classification.

Subject	Number of data					Accuracy	F1 score
	0	1	2	3	Total		
1	7	0	0	0	7	100.00	100.00
2	7	7	7	29	50	100.00	100.00
3	0	29	7	0	36	97.22	95.31
4	0	7	7	0	14	100.00	100.00
5	0	0	7	0	7	100.00	100.00
6	0	14	29	0	43	81.40	80.89
7	0	7	7	7	21	90.48	90.28
8	0	29	7	0	36	91.67	83.90
9	0	14	22	7	43	97.67	98.08
10	0	14	7	0	21	100.00	100.00
11	7	14	0	29	50	88.00	86.00
12	0	0	7	21	28	85.71	83.63
13	0	7	0	36	43	100.00	100.00
14	0	7	14	0	21	95.24	94.43
15	7	21	22	7	57	91.23	89.26
16	0	7	0	14	21	100.00	100.00
17	0	50	22	0	72	98.61	98.34
18	0	36	0	0	36	100.00	100.00
19	0	7	0	0	7	100.00	100.00
20	0	14	29	0	43	81.40	78.82
21	0	14	36	0	50	98.00	97.46
22	0	29	0	14	43	95.35	94.49
23	14	0	0	0	14	100.00	100.00
24	0	7	57	22	86	87.21	84.41
26	0	36	0	0	36	100.00	100.00
29	0	7	0	29	36	100.00	100.00
30	0	29	14	0	43	90.70	90.05
31	7	21	0	36	64	92.19	93.33
Total	49	427	301	251	1028		
Average						95.07	94.24

Accuracy and F1 score are shown as a percentage.

The LOSO result that input validation data instead of test data into the trained model was an accuracy of 99.34% and an F1 score of 99.34%. This result shows that the low accuracy of the test data in LOSO is not because the model does not fit the training data well enough but because the test subjects’ data has different characteristics from other subjects’ data (the training data). The 40-channel fear physiological signal in the DEAP dataset used in this paper can differ significantly in individuals. Further verification is required to determine whether these individual differences are attributed to 32-channel EEG or 8-channel PPS. Channel selection with high relevance to fear could be an excellent solution to avoid individual differences and improve LOSO accuracy.

**Figure 6:**
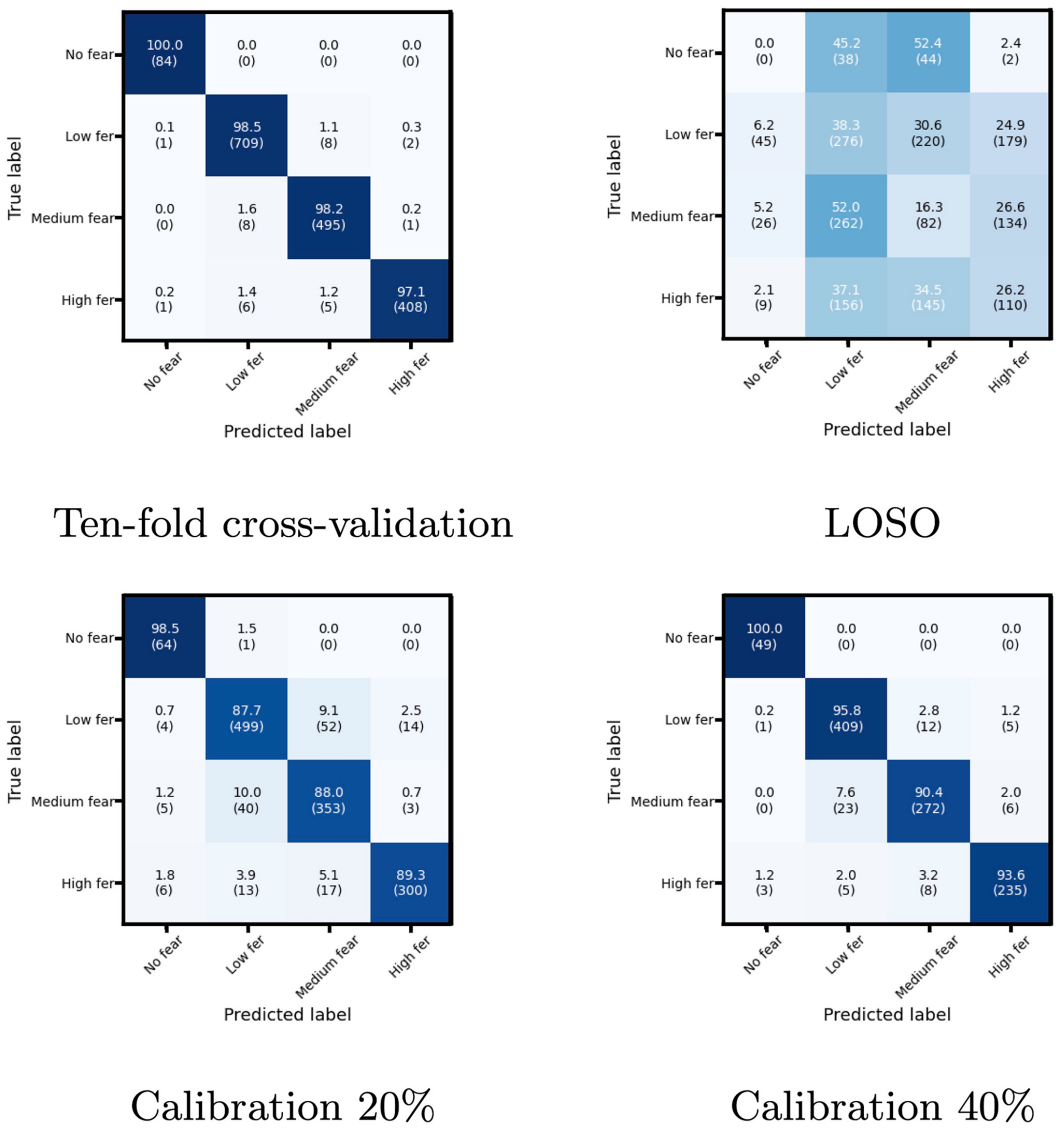
The confusion matrix of the four evaluation methods. The vertical axis is the true label, and the horizontal axis is the prediction label. The diagonal values represent the recall for each class, and the sum of the row is 100%. The contents of the parentheses indicate the number of data.

Figure 6 shows the confusion matrix of the four evaluation methods. Even though the proposed model achieved higher classification accuracy than the other studies in the 10-fold cross-validation, many misclassifications occurred in LOSO. From another point of view, the proposed model cannot accommodate the characteristics of individual differences the DEAP dataset has. While Calibration succeeded in reducing misclassification, the misclassification between Low fear and Medium fear caused by the data characteristics was noticeable. This tendency is also true for the 10-fold cross-validation. Discovering a more expressive model structure that can adequately accommodate individual differences will also be a fundamentally important problem.

Because the DEAP is not a fear-specific emotion database, not all subjects have data for all fear levels. In particular, only six have data of No fear (see Table 6). In LOSO of Figure 6, No fear has 0% accuracy, and others also have low accuracy. These indicate that the poor number of subjects for each level leads to low recognition accuracy in LOSO. The model would accommodate individual differences by using a database with a sufficient number of users per level. Such a database would be helpful to classify fear in more than four levels.

This paper also checked the LOSO accuracy of two-level classification with fear (1 < =valence<=5, 5 < =arousal<=9, 1 < =dominance<=5) and without fear (5 < valence<=9, 1 < =arousal<5, 5 < =dominance<9) and resulted in an accuracy of 51.02% and an F1 score of 41.42%. Table 9 shows the number of test data, accuracy, and F1 score per test subject in LOSO for two-level classification. The two-level is low accuracy despite having more subjects and data than the four-level. The fear level in this paper is defined based on the self-assessment score in the DEAP. This result suggests that subjects in the DEAP dataset may not correctly assess their fear level.

**Table 9:** The number of test data, accuracy, and F1 score per test subject in LOSO for two-level classification.

Subject	Number of data			Accuracy	F1 score
	0	1	Total		
1	72	48	120	53.33	49.76
2	60	84	144	68.75	64.66
3	120	12	132	62.88	40.46
4	12	48	60	76.67	54.25
5	0	12	12	0.00	0.00
6	132	48	180	36.67	34.03
7	36	24	60	58.33	54.70
8	60	48	108	39.81	39.81
9	24	84	108	50.00	49.72
10	60	72	132	51.52	50.38
11	180	60	240	26.67	24.35
12	0	156	156	10.90	9.83
13	24	108	132	15.15	13.16
14	72	120	192	54.69	50.64
15	132	132	264	46.97	42.96
16	48	96	144	54.86	46.47
17	96	36	132	42.42	35.23
18	72	24	96	72.92	42.17
19	12	12	24	66.67	62.50
20	36	132	168	22.62	22.58
21	24	120	144	19.44	18.17
22	60	120	180	51.11	47.70
23	132	0	132	90.15	47.41
24	48	180	228	37.72	36.62
25	48	36	84	71.43	65.00
26	120	12	132	76.52	43.35
27	72	0	72	48.61	32.71
28	84	0	84	73.81	42.47
29	12	60	72	68.06	60.79
30	96	24	120	67.50	57.14
31	156	144	300	52.00	34.21
32	108	36	144	64.58	52.33
Total	2,208	2088	4296		
Average				51.02	41.42

Accuracy and F1 score are shown as a percentage.

The dataset used in this study was previously processed by removing the eye artifact and baseline, applying a bandpass frequency filter from 4.0 to 45.0 Hz, and averaging over the common reference. This paper added standardization to the dataset. The LOSO adopting normalization instead of standardization improved the accuracy to 41.49% and the F1 score to 25.27%. Standardization probably could have been more effective if there were no large-scale differences between the training and test data. Data preprocessing to compensate for differences between individuals would provide room for improvement in accuracy. Ahmed et al. (2023) improved the LOSO classification accuracy by removing the baseline from the EEG signals in the DEAP dataset using an original method to correct for individual differences in the data. The 10-fold cross-validation result without standardization was an accuracy of 98.84% and an F1 score of 98.95%. This paper achieved high classification accuracy with applying only minimal preprocessing by building a model that suited the data.

## 5. Conclusion

This paper succeeded in estimating human fear in four levels (No fear, Low fear, Medium fear, and High fear) using multichannel EEG and multimodal peripheral physiological signals stored in the DEAP dataset with an accuracy of 98.79% and an F1 score of 99.01% higher than the previous studies’ method in 10-fold cross-validation. The first contribution of this paper was to demonstrate the possibility of recognizing the fear emotion with high accuracy using a Deep Learning model from physiological signals with minimal preprocessing without arbitrary feature extraction or feature selection. The second contribution was to discover an effective model structure for fear emotion recognition, in which EEG and peripheral physiological signals are separately input to the CNN for feature extraction and then input to the LSTM. As the third contribution, this paper evaluated that the proposed model could cope with the characteristics of individual differences in physiological signals through a reasonable volume of additional learning. This paper’s limitation is examining whether the fear level is dividable into further details. DEAP dataset is not specific to the fear emotions, and a four-level classification was appropriate from the viewpoint of the number of samples per fear level. Fear-specific datasets with sufficient samples for each detailed level are indispensable for developing better Deep Learning fear estimation models. We should collect more and more fear data from Electroencephalography and annotate fear levels for machine learning labels.

## Data availability statement

Publicly available datasets were analyzed in this study. This data can be found here: The datasets analyzed for this study can be found in DEAP dataset: http://www.eecs.qmul.ac.uk/mmv/datasets/deap/readme.html.

## Author contributions

NM was responsible for data processing and data analysis. IY reviewed and edited the manuscript. All authors contributed to study design, manuscript writing, and approved the submitted version.

## Funding

This work was supported by Grants-in-Aid for Scientific Research (B) 20H04476 from Japan Society for the Promotion of Science (JSPS).

## Conflict of interest

The authors declare that the research was conducted in the absence of any commercial or financial relationships that could be construed as a potential conflict of interest.

## Publisher’s note

All claims expressed in this article are solely those of the authors and do not necessarily represent those of their affiliated organizations, or those of the publisher, the editors and the reviewers. Any product that may be evaluated in this article, or claim that may be made by its manufacturer, is not guaranteed or endorsed by the publisher.
